# Inhibition of xanthine oxidoreductase with febuxostat, but not allopurinol, prevents inflammasome assembly and IL-1β release

**DOI:** 10.26508/lsa.202403191

**Published:** 2025-05-21

**Authors:** Lauar de Brito Monteiro, Anne-Sophie Archambault, Lucas F Starchuk, Armando Alcazar, Ju Hee Oh, Joshua A Dubland, Bojana Rakić, Annette E Patterson, C Bruce Verchere, Ramon I Klein Geltink

**Affiliations:** 1 BC Children’s Hospital Research Institute, Vancouver, Canada; 2 University of British Columbia , Department of Surgery, Vancouver, Canada; 3 University of British Columbia , Department of Pathology and Laboratory Medicine, Vancouver, Canada; 4 University of British Columbia , Department of Biochemistry and Molecular Biology, Vancouver, Canada; 5 University of British Columbia , Centre for Molecular Medicine and Therapeutics, Vancouver, Canada; 6 University of British Columbia , Edwin S.H. Leong Centre for Healthy Aging, Vancouver, Canada; 7 BC Cancer Research Centre, Department of Basic and Translational Research, Vancouver, Canada

## Abstract

Xanthine oxidoreductase (XOR) inhibitors are used to treat gout, inhibiting uric acid production, which causes clinical symptoms. Commonly used XOR inhibitors are the small molecule febuxostat (Fbx) and the purine analogue allopurinol (Allo). Recent studies show that XOR inhibitors can reduce mature interleukin (IL)-1β production by activated macrophages. This effect is not due to reduced uric acid crystal formation, which can induce NLRP3 inflammasome activation, but an independent effect. Fbx and Allo have been used interchangeably in in vitro studies to highlight the role of XOR in pro-inflammatory macrophage function. Here, we analysed the effects of Fbx and Allo on pro-inflammatory macrophage signatures. Both XOR inhibitors maintain pro-inflammatory macrophage metabolic and phenotypic hallmarks. However, only Fbx reduces the activity of caspase-1 and the release of IL-1β by preventing inflammasome assembly in macrophages isolated from both mice and humans. Our study identified an Fbx-specific reduction in IL-1β production, which could be used clinically to reduce the deleterious effects of macrophage-derived IL-1β.

## Abstract (precis)

This study focuses on the underappreciated distinct effects of two clinically used xanthine oxidoreductase inhibitors: allopurinol and febuxostat. Besides reducing uric acid levels in gout treatment, febuxostat, but not allopurinol, prevents NLRP3 inflammasome assembly and blocks IL-1β production by macrophages.

## Introduction

Xanthine oxidoreductase (XOR) is an enzyme catalysing the final steps of the purine catabolism pathway, responsible for the degradation of hypoxanthine and xanthine to uric acid (Sekizuka, 2022). Hyperuricaemia (elevated levels of uric acid) can occur as a result of not only excessive purine degradation, for example, during haemolysis or tumour lysis syndrome, but also dietary purines. Hyperuricaemia increases the risk of developing gout and associated morbidities, such as nephropathy (Becker et al, 2005; George et al, 2024). Currently, the best described therapy to manage hyperuricaemia disorders involves inhibition of XOR. Clinically approved XOR inhibitors include febuxostat (Fbx), a small molecule that is a specific, non-competitive XOR inhibitor (Becker et al, 2005; Battelli et al, 2014), and allopurinol (Allo), which is a purine analogue that competitively limits XOR activity. Although Fbx has been shown to be more effective than Allo in lowering urate levels in the clinic (Becker et al, 2005), these drugs are interchangeably referred to as efficient XOR inhibitors.

Macrophage metabolism has been extensively studied in the last few decades. It is intrinsically connected to their plasticity and pro-inflammatory function (Kelly & O’Neill, 2015; Van den Bossche et al, 2017). Macrophages are a major source of the pro-inflammatory cytokine interleukin (IL)-1β. The release of mature IL-1β is dependent on pro-IL-1β cleavage by caspase-1 after NLRP3 inflammasome assembly (Lopez-Castejon & Brough, 2011).

Major inflammatory reactions in gout are associated with recruitment of innate immune cells, some of which respond to monosodium urate (MSU) crystals, triggering NLRP3 inflammasome activation (Zhao et al, 2022). It was hypothesized that blockade of XOR to reduce urate levels could dampen the release and effects of macrophage-derived IL-1β (Lopez-Castejon & Brough, 2011; Nomura et al, 2019). Previous reports have shown that XOR inhibition–mediated reduction of IL-1β levels is independent of changes in uric acid–mediated NLRP3 activation (Ives et al, 2015). XOR is a source of reactive oxygen species (ROS), as well as xanthine and uric acid, a reaction that occurs in the cytosol, but is also associated with an increase in mitochondrial ROS, contributing to NLRP3 activation and the secretion of the mature form of IL-1β (Ives et al, 2015). These studies point to possible differences regarding the effects of Fbx and Allo in macrophages. Allo being a purine analogue inhibits XOR and hypoxanthine phosphoribosyltransferase (HPRT) (Hosoya et al, 2020), whereas Fbx is XOR-specific (Nomura et al, 2019). In addition, during NLRP3 induction with nigericin, Fbx treatment can maintain intracellular ATP levels through purine salvage pathway activation, which was not the case with Allo-treated macrophages (Nomura et al, 2019). Here, we explored the distinct effects of Fbx- and Allo-mediated XOR inhibition in mouse and human macrophages, identifying that XOR inhibition by Fbx, but not Allo, inhibits assembly of the NLRP3 inflammasome.

## Results and Discussion

### Distinct effects of febuxostat and allopurinol in macrophage pro-inflammatory phenotype

**Figure 1.**
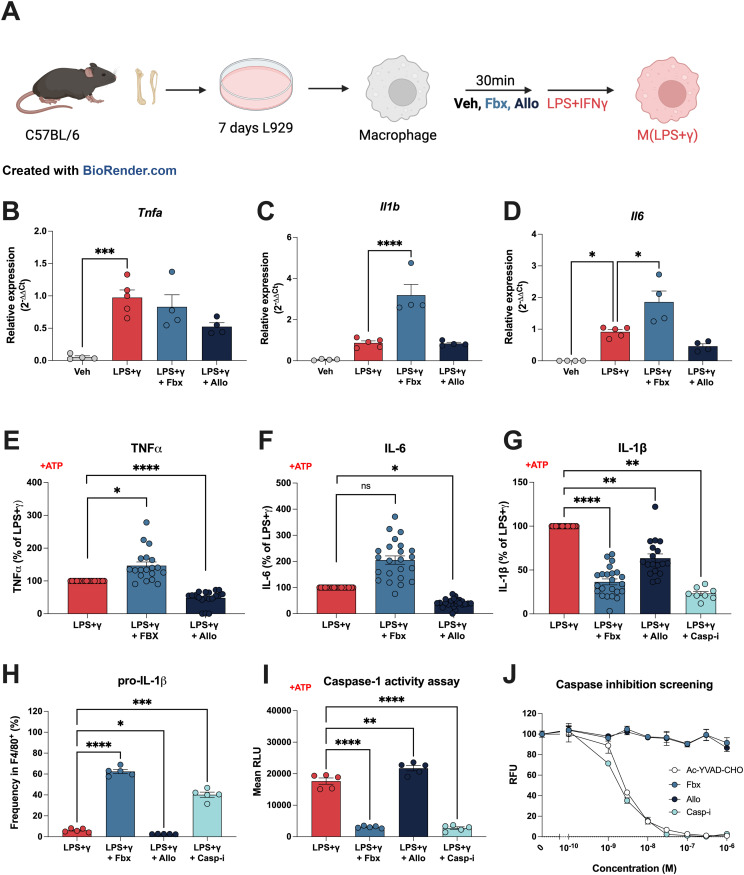
Differential regulation of macrophage inflammation by Fbx and Allo. **(B, C, D, E, F, G, H, I)** Mouse bone marrow–derived macrophages were pre-treated for 30 min with DMSO vehicle (Veh), Fbx (200 µM), Allo (250 μg/ml), or Casp-i (Z-YVAD-FMK, 20 μM), and then with LPS (100 ng/ml) and IFNγ (20 ng/ml) for 6 h (B, C, D, E, F, G, H, I) or 18 h (H). (E, F, G, H, I) In experiments in which cytokine release was measured (E, F, G, H), ATP (1 mM) was added 3 h before harvesting (E, F, G, H, I). **(A)** Schematic representation of the macrophage differentiation and polarization protocol. **(B, C, D)** *Tnfa*, *Il6*, and *Il1b* gene expression measured by qRT-PCR. **(E, F, G)** TNF-ɑ, IL-6, and IL-1β protein levels were measured by ELISA. Results are represented as the percentage of M(LPS+γ). **(H)** pro-IL1β frequency in F4/80^+^ cells determined by flow cytometry. **(I)** Caspase-1 activity (relative luminescence units, RLU) was measured according to the manufacturer’s protocol. Results are represented as the percentage of M(LPS+γ). **(J)** Caspase inhibition screening test (relative fluorescence units, RFU). Caspase-1 inhibitor Ac-YVAD-CHO was used as a control, according to the manufacturer’s instructions. Results are the mean ± SEM of at least five biological replicates. *P*-values were calculated (one-way ANOVA) on the data before normalization. *P < 0.05; **P < 0.01; ***P < 0.001; ****P < 0.0001.

**Figure S1.**
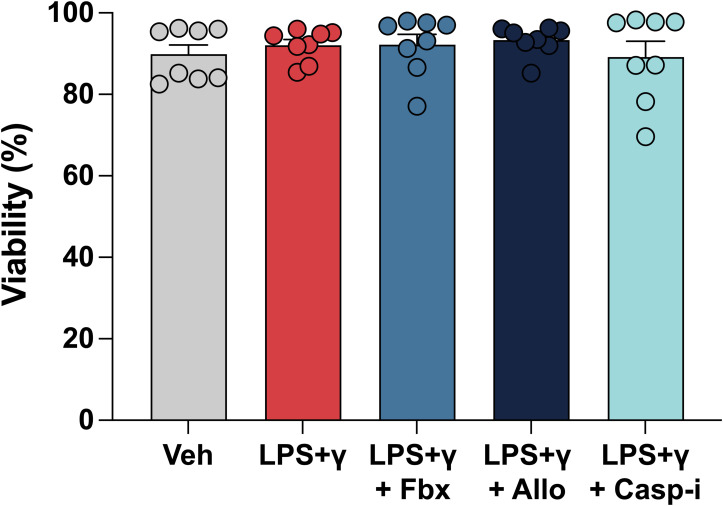
Impact of Fbx and Allo on cellular viability. Mouse bone marrow–derived macrophages were pre-treated for 30 min with DMSO vehicle (Veh), Fbx (200 µM), Allo (250 μg/ml), or Casp-i (Z-YVAD-FMK, 20 μM), followed by polarization with LPS (100 ng/ml) and IFNγ (20 ng/ml) for 6 h. Frequency of live cells by flow cytometry. Results are representative of at least four biological replicates.

Two recent studies show inhibitors of purine catabolism as efficient inhibitors of NLRP3 activity (Ives et al, 2015; Nomura et al, 2019). Given the distinct nature of these clinically used XOR inhibitors, we asked whether these drugs could be used interchangeably in the alteration of macrophage-derived pro-inflammatory signals (Fig 1A). We used murine bone marrow–derived macrophages (BMDMs) polarized towards a pro-inflammatory phenotype by LPS and interferon gamma (IFNγ) [M(LPS+γ)] in vitro. M(LPS+γ) increased the mRNA expression of the pro-inflammatory cytokines *Tnfa*, *Il6*, and *Il1b* (Fig 1B–D). Pre-treatment with Fbx or Allo did not alter cellular viability (Fig S1), nor the transcriptional up-regulation of these pro-inflammatory genes by LPS+γ, whereas Fbx-treated BMDMs showed an increase in the expression of *Il6* and *Il1b* compared with M(LPS+γ) (Fig 1B–D). In agreement with the up-regulated mRNA, only Fbx-treated BMDMs increased secretion of TNF-α and IL-6 compared with vehicle-treated cells (Fig 1E and F).

**Figure S2.**
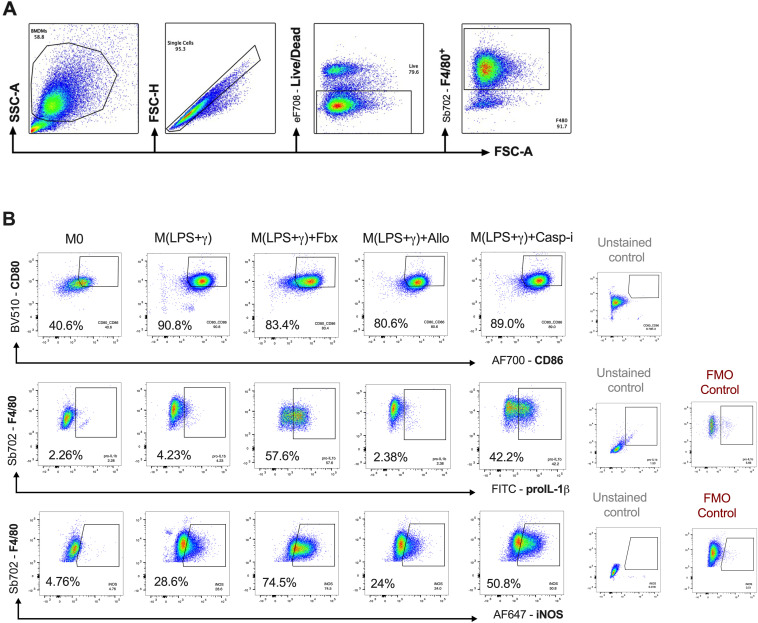
Flow cytometry gating strategy. **(A)** Gating strategy to identify F4/80^+^ BMDMs. **(B)** Gating strategy showing CD86^+^, pro-IL-1β^+^, and iNOS^+^ BMDMs from F4/80^+^ gate pre-treated for 30 min with DMSO vehicle (Veh), Fbx (200 µM), Allo (250 μg/ml), or Casp-i (Z-YVAD-FMK, 20 μM), and then with LPS (100 ng/ml) and IFNγ (20 ng/ml) for 18 h.

Compared with vehicle-treated cells, pre-treatment of BMDMs with either XOR inhibitor dampened IL-1β secretion 3 h after the addition of the NLRP3 activator ATP (Fig 1G), but Fbx-treated BMDMs showed lower levels of secreted IL-1β compared with Allo-treated cells (*P* = 0.0011) (Fig 1G). Because IL-1β is secreted in its caspase-1–cleaved mature form after assembly and activation of the NLRP3 inflammasome (Lopez-Castejon & Brough, 2011), we next asked whether caspase-1 activity was perturbed upon inhibition of XOR and included the caspase-1 inhibitor Z-YVAD-FMK (Casp-i) as a positive control to block secretion of the mature form of IL-1β (Fig 1G). Intracellular staining and flow cytometry analysis revealed that the reduced caspase-1 activity also led to pro-IL-1β accumulation in BMDMs treated with Fbx and Casp-i, but not after treatment with Allo (Figs 1H and S2A and B). In addition, BMDMs treated with Fbx showed significantly reduced caspase-1 activity—similar to cells treated with Casp-i—whereas Allo-treated BMDMs maintained caspase-1 activity comparable to vehicle-treated cells (Fig 1I). To examine whether caspase-1 activity and secretion of IL-1β in BMDMs were due to a potential off-target effect of Fbx directly inhibiting caspase-1, we performed an in vitro caspase-1 inhibition screening using purified caspase-1. These results clearly showed that neither Fbx nor Allo is a direct inhibitor of caspase-1, when compared to known caspase-1 inhibitor controls (Fig 1J). Together, these results demonstrate that Fbx and Allo have different effects on the secretion of pro-inflammatory cytokines, with Fbx but not Allo causing a drastic reduction in caspase-1 activity without directly inhibiting caspase-1.

### Febuxostat and allopurinol treatment maintains macrophage pro-inflammatory signature

**Figure 2.**
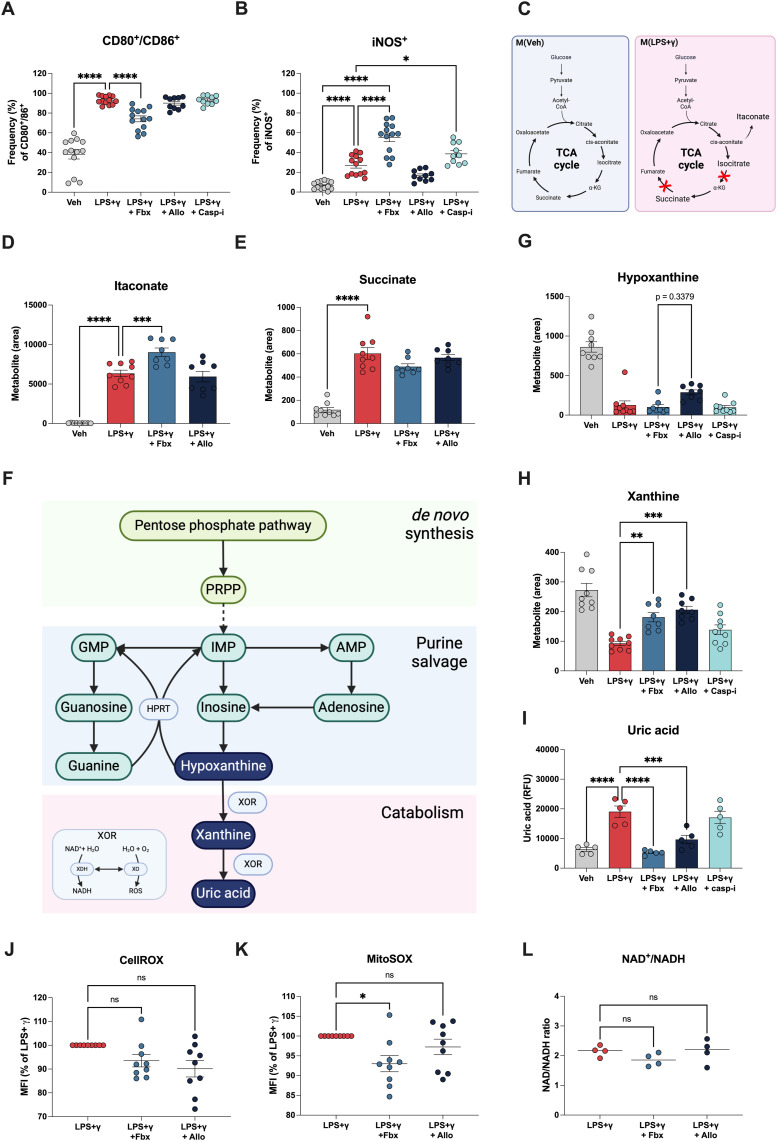
Fbx and Allo treatments preserve macrophage inflammatory features. **(A, B, C, D, E, F, G, H, I, J, K, L)** Mouse bone marrow–derived macrophages were pre-treated for 30 min with DMSO vehicle (Veh), Fbx (200 µM), Allo (250 μg/ml), or Casp-i (Z-YVAD-FMK, 20 μM), followed by polarization with LPS (100 ng/ml) and IFNγ (20 ng/ml) for 18 h (A, B, C, D, E, F, G, H, I) or 2 h (J, K, L). **(A)** Frequency of CD80 and CD86 double-positive macrophages determined by flow cytometry. **(B)** Frequency of iNOS^+^ macrophages by flow cytometry. **(C)** Schematic diagram representing breaks in TCA cycle promoted by LPS+γ polarization of macrophages. **(D, E)** Peak area comparison of itaconate and succinate obtained by liquid chromatography–mass spectrometry (LC-MS). **(F)** Schematic representation of the purine pathway. **(G, H)** Peak area comparison of hypoxanthine and xanthine obtained by liquid chromatography–mass spectrometry (LC-MS). **(I)** Uric acid levels were measured with a colorimetric assay according to the manufacturer’s instructions. **(J, K)** Cellular ROS and mitochondrial superoxide were determined using the probes CellROX and MitoSOX, respectively. Fluorescence was measured by flow cytometry. **(L)** NAD+ and NADH levels were measured using a luminescence assay according to the manufacturer’s instructions. Data are represented as the NAD/NADH ratio. Results are the mean ± SEM of at least three biological replicates. *P*-values were calculated (one-way ANOVA). * *P* < 0.05; **P < 0.01; ***P < 0.001; ****P < 0.0001.

Macrophages are plastic cells, influenced by many extracellular factors; changes in macrophage function are often inferred from changes in phenotypic markers (Shapouri-Moghaddam et al, 2018; Locati et al, 2020). To characterize changes in macrophage phenotype associated with clinically approved XOR inhibitors, we analysed the expression of pro-inflammatory markers in live non-polarized and M(LPS+γ) BMDMs (F4/80^+^) by flow cytometry after treatment with Fbx, Allo, or Casp-i. M(LPS+γ) BMDMs increased the expression of CD80/CD86, co-stimulatory surface molecules that are promptly up-regulated by pro-inflammatory signalling. Fbx treatment mildly reduced the frequency of CD80^+^/CD86^+^ cells compared with M(LPS+γ) treatment alone, but not to basal levels observed in non-polarized (vehicle-treated) BMDMs (Figs 2A and S2A and B). Allo and Casp-i did not induce any changes in CD80^+^/CD86^+^ frequency compared with M(LPS+γ) alone (Fig 2A). Inducible nitric oxide synthase (iNOS) is an enzyme that catalyses nitric oxide (NO) production as a defence mechanism during inflammation. The frequency of iNOS-expressing cells was significantly up-regulated after Fbx and Casp-i treatment in M(LPS+γ), whereas Allo did not increase iNOS expression beyond the levels observed in vehicle-treated M(LPS+γ) cells (Fig 2B). These data show that besides a slight reduction in CD80^+^/CD86^+^ frequency, Fbx induces a comparable macrophage activation phenotype to that observed with specific Casp-i, in contrast to cells treated with Allo.

Another hallmark of M(LPS+γ) macrophages is a “broken” tricarboxylic acid (TCA) cycle with accumulation of intermediary metabolites (Jha et al, 2015). Accumulation of isocitrate, itaconate, and succinate is hallmark of M(LPS+γ) macrophages and functionally important for pro-inflammatory macrophage function, as these metabolites act as regulators of inflammation and defence against microorganisms (Jha et al, 2015; Liu et al, 2021; O’Neill & Artyomov, 2019) (Fig 2C). Treatment of M(LPS+γ) BMDMs with Fbx or Allo did not alter the accumulation of the TCA intermediates itaconate and succinate (Fig 2D and E). These results show that although inhibition of XOR with either Fbx or Allo alters the secretion of pro-inflammatory cytokines, XOR inhibition does not affect the typical pro-inflammatory hallmarks of M(LPS+γ) BMDMs.

To better understand the difference between the effects of macrophage XOR inhibition by Fbx and Allo, we measured levels of purines by LC-MS/MS-based metabolomics. Purine levels are regulated by the balance of de novo synthesis, salvage, and catabolism (Pedley & Benkovic, 2017) (Fig 2F). Salvage is used as a way to regenerate AMP, IMP, GMP. These purine ribonucleotides are essential for biosynthesis of biomolecules (DNA synthesis, protein synthesis, etc.). Hypoxanthine can be generated from inosine, and salvaged by HPRT to generate IMP or GMP (Pedley & Benkovic, 2017). Hypoxanthine can be catabolized to the irreversible metabolite xanthine and then to uric acid, both reactions catalysed by XOR (Bortolotti et al, 2021). When we compared purine levels in BMDMs treated with Fbx or Allo, our data suggest that hypoxanthine accumulated in Allo-treated cells but not in Fbx-treated cells (non-significant; *P* = 0.2809) (Fig 2G); however, a similar increase in xanthine levels was observed in Fbx- and Allo-treated BMDMs (Fig 2H). This is in support of previous reports showing HPRT inhibition by Allo, but not Fbx (Tani et al, 2019; Hosoya et al, 2020). Both drugs, but not the Casp-i control, reduced uric acid levels, in line with their clinical use as XOR inhibitors in patients suffering from uricaemia (Fig 2I).

In mammals, XOR has relatively low substrate specificity and can undergo post-translational modifications that allow for various physiological functions (Terao et al, 2020; Bortolotti et al, 2021). XOR can generate NADH, reactive oxygen species (ROS), or NO, not only highlighting XOR as a critical enzyme in purine catabolism, but also impacting cellular redox state and defence against microorganisms. Previous reports have shown that reduction of IL-1β levels post-XOR inhibition happens because of a urate-independent mechanism (Ives et al, 2015). XOR is a source of both cytoplasmic and mitochondrial ROS during inflammation (Battelli et al, 2014), as XOR is localized in both intracellular compartments and has been reported to contribute to NLRP3 activation and the secretion of mature form IL-1β (Ives et al, 2015). Our findings revealed that after 2 h of activation, there was no change in the cellular ROS accumulation, measured by the probe CellROX (Fig 2J), or in the NAD+/NADH ratios between all conditions (Fig 2L). Using the probe MitoSOX, we found that Fbx treatment reduced the accumulation of mitochondrial ROS only at 2 h post-stimulation, but no significant changes were observed after Allo treatment (Fig 2K). Although these data corroborate a previous study that showed reduced mitochondrial ROS after Fbx-mediated XOR inhibition, the unchanged mitochondrial ROS levels after Allo treatment suggest that only Fbx-mediated XOR inhibition reduces mitochondrial ROS production.

### Blockade of mature IL-1β release by Fbx is conserved in human macrophages

**Figure 3.**
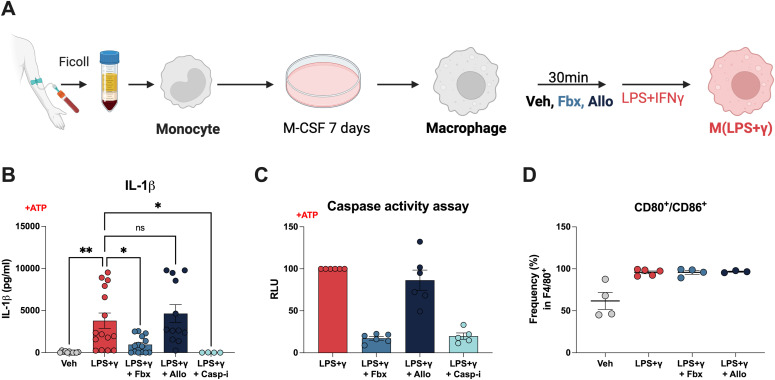
Differential impact of Fbx and Allo on macrophage inflammatory profile is conserved in human macrophages. **(B, C, D)** Human monocyte-derived macrophages were pre-treated for 30 min with DMSO vehicle (Veh), Fbx (200 µM), Allo (250 μg/ml), or Casp-i (Z-YVAD-FMK, 20 μM), and then with LPS (100 ng/ml) and IFNγ (20 ng/ml) for 6 h (B, C) or 18 h (D). **(B, C)** ATP (1 mM) was added 3 h before harvesting. **(A)** Schematic representation of the differentiation of monocyte-derived macrophages. **(B)** IL-1β levels measured by ELISA. **(C)** Caspase-1 activity (relative luminescence units, RLU) was measured according to the manufacturer’s protocol. **(D)** Frequency of CD80 and CD86 double-positive macrophages determined by flow cytometry. Results are the mean ± SEM of at least four biological replicates. *P*-values were calculated using one-way ANOVA. * *P* < 0.05; **P < 0.01.

**Figure S3.**
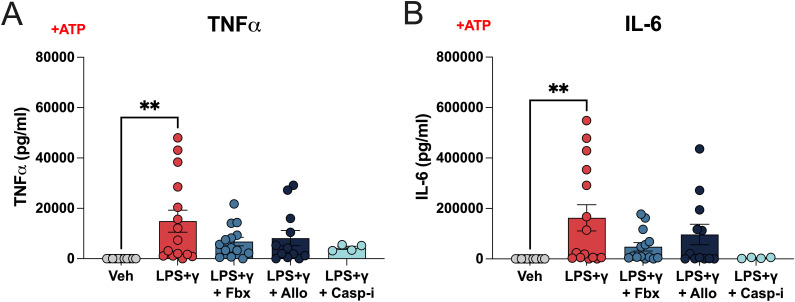
Impact of Fbx and Allo on TNF-ɑ and IL-6 secretion in human macrophages. Human monocyte-derived macrophages were pre-treated for 30 min with DMSO vehicle (Veh), Fbx (200 µM), Allo (250 μg/ml), or Casp-i (Z-YVAD-FMK, 20 μM), and then with LPS (100 ng/ml) and IFNγ (20 ng/ml) for 6 h. ATP (1 mM) was added 3 h before harvesting. **(A)** TNF-α levels measured by ELISA. **(B)** IL-6 levels measured by ELISA. *P*-values were calculated using one-way ANOVA. **P < 0.01.

In a clinical study that recruited 80 patients with gout, Fbx was shown to reduce the circulating levels of IL-1 compared with Allo (Hao et al, 2019). Therefore, we assessed the effect of XOR inhibition in human monocyte-derived macrophages (MDMs). As in BMDM experiments, MDMs were pre-treated for 30 min with Fbx or Allo, followed by treatment with LPS+IFNγ (Fig 3A). We found that similar to mouse BMDMs, Fbx pre-treatment reduced mature IL-1β release post–pro-inflammatory polarization, which did not occur in Allo-treated MDMs (Fig 3B). Fbx—but not Allo—also consistently reduced caspase-1 activity (Fig 3C). We did not observe a statistically significant increase in TNF-α and IL-6 secretion in Fbx-treated MDMs (Fig S3A and B). In humans, macrophage activation with LPS+IFNγ was sufficient to sustain high secretion of TNF-α and IL-6 after Fbx, Allo, or Casp-i treatments (Fig S3A and B). Similar to our observation in mouse BMDMs, pre-treatment of MDMs with Fbx or Allo did not alter the pro-inflammatory phenotypic signature of human macrophages, with the frequency of CD80^+^/CD86^+^ cells comparable to M(LPS+γ) (Fig 3D). Combined, these data suggest that Fbx-mediated XOR inhibition, but not XOR inhibition with Allo, blocks NLRP3/caspase-1 inflammasome activation in both mice and humans.

### Fbx prevents inflammasome assembly in pro-inflammatory macrophages

**Figure 4.**
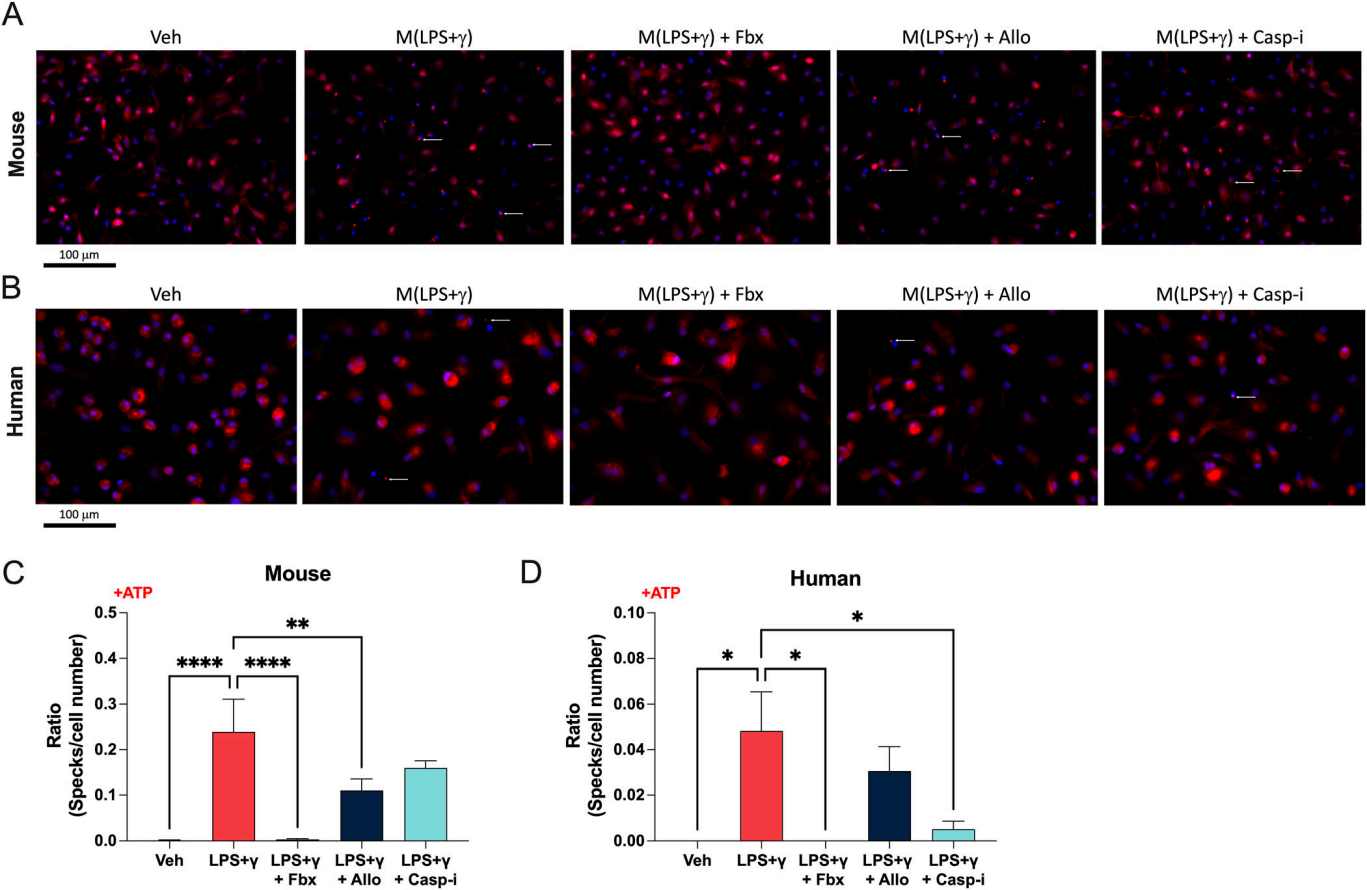
Fbx inhibits inflammasome assembly. Mouse bone marrow–derived macrophages or human monocyte-derived macrophages were pre-treated for 30 min with DMSO vehicle (Veh), Fbx (200 µM), Allo (250 μg/ml), or Casp-i (Z-YVAD-FMK, 20 μM), followed by polarization with LPS (100 ng/ml) and IFNγ (20 ng/ml) for 6 h. **(A, B, C, D)** ATP (1 mM) was added 3 h before harvesting (A, B, C, D). **(A)** Fluorescence microscopy of ASC1 (red), DAPI (blue) in bone marrow–derived macrophages. ASC specks are indicated by white arrows. **(B)** Fluorescence microscopy of ASC1 (red), DAPI (blue) in human monocyte-derived macrophages. **(C)** Average number of detected specks per cell in mouse samples. **(D)** Average number of detected specks per cell in human samples. **(C, D)** Results are represented as total number of specks divided by the total number of cells per sample. No more than one speck per cell was observed. Results are representative of at least three biological replicates.

**Figure S4.**
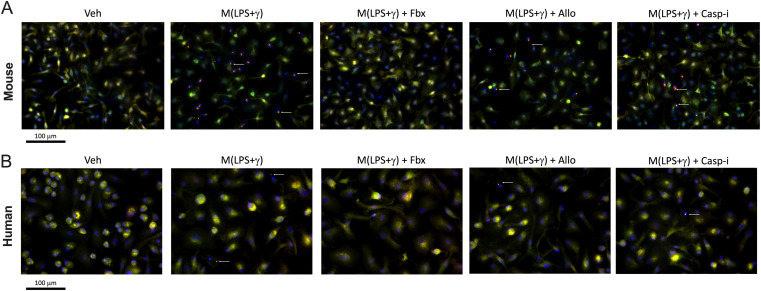
Fbx inhibits inflammasome assembly. Mouse bone marrow–derived macrophages or human monocyte-derived macrophages were pre-treated for 30 min with DMSO vehicle (Veh), Fbx (200 µM), Allo (250 μg/ml), or Casp-i (Z-YVAD-FMK, 20 μM), followed by polarization with LPS (100 ng/ml) and IFNγ (20 ng/ml) for 6 h. ATP (1 mM) was added 3 h before harvesting. **(A)** Fluorescence microscopy of ASC1 (red), NLRP3 (green), and DAPI (blue) in bone marrow–derived macrophages. ASC specks are indicated by white arrows. **(B)** Fluorescence microscopy of ASC1 (red), NLRP3 (green), and DAPI (blue) in human monocyte-derived macrophages. Results are representative of at least three biological replicates.

The inflammasome is a multi-protein complex responsible for caspase-1 activation. The assembly of this large protein complex is dependent on the caspase recruitment domain (CARD) in the apoptosis-associated speck-like protein containing CARD (ASC), in the large NLRP3 inflammasome complex filaments (Sborgi et al, 2015). When the NLRP3 inflammasome is activated, ASC filaments aggregate and become visible in the form of a large intracellular “speck,” which can be used as a readout for inflammasome activation and assembly (Masumoto et al, 1999; Stutz et al, 2013). We stained mouse BMDMs using anti-ASC and anti-NLRP3 antibodies and performed fluorescence microscopy to analyse “speck-like” formations. ASC signal is present diffusely throughout the cell in resting macrophages (Figs 4A and S4A and B). In M(LPS+γ) BMDMs, clear specks are visible, but strikingly, Fbx pre-treatment prevented the formation of specks in M(LPS+γ), which were still visible after either Allo or caspase-1 inhibition (Fig 4A and C). In human MDMs, we could readily detect ASC speck formations after LPS+γ treatment in cells from all individual healthy control donors (Fig 4B and D). XOR inhibition by Fbx prevented speck formation in pro-inflammatory human MDMs, whereas Allo and Casp-i did not (Fig 4B and D). Our data show that Fbx-dependent blockade of ASC speck formation is conserved in both mice and humans and that Fbx-induced XOR inhibition is crucial for NLRP3 inflammasome assembly.

Taken together, our data indicate that XOR inhibition impacts pro-inflammatory macrophage function without major alterations to widely used pro-inflammatory macrophage phenotypic markers. Our results further show that Fbx-sensitive XOR activity directly supports inflammasome assembly, because the inhibition of XOR with Fbx leads to a reduction in caspase-1 activity, resulting in an accumulation of intracellular pro-IL-1β which can no longer be cleaved and released as mature IL-1β.

In summary, we show the distinct effects of two clinically used XOR inhibitors, Fbx and Allo, on macrophage pro-inflammatory function. Fbx blocked NLRP3 inflammasome assembly as indicated by a loss of ASC speck formation. This, in turn, results in lower caspase-1 activity and inhibits the conversion of pro-IL-1β into its mature form. Although both XOR inhibitors efficiently reduce uric acid levels and are used in the treatment of gout and arthritis, this study is the first to show a potential use for Fbx-mediated XOR inhibition in the treatment of inflammatory diseases mediated by NLRP3 inflammasome activity and macrophage-derived IL-1β.

## Materials and Methods

### Mice

C57BL/6 mice were purchased from the Jackson laboratory and kept at the BC Children’s Hospital Research Institute Animal Facility (protocol #A23-0282), in compliance with applicable guidelines. Female and male mice aged 12–20 wk were used for experiments.

### L929 media

L929 cells were seeded and cultured in RPMI (#10-040-CV; Corning) containing 10% FBS (#12483020; Thermo Fisher Scientific), 10 mM glutamine (#25030164; Thermo Fisher Scientific), 1% Pen/Strep (#SV30010; Thermo Fisher Scientific) for 10 d. Media were harvested, residual cells were removed by centrifugation, and media were filtered. L929 media were then tested for bone marrow–derived macrophage differentiation efficiency by flow cytometry, as described below.

### Bone marrow–derived macrophages

Bone marrow was collected from C57BL/6 mice. Red blood cells were lysed with red blood cell lysis buffer (150 mM ammonium chloride, 10 mM potassium bicarbonate, 0.1 mM EDTA, pH 7.3–7.4) for 3 min, and residual cells were plated in RPMI containing 10% FBS, 1% Pen/Strep, 1% HEPES (#SH3023701; Thermo Fisher Scientific), and 20% L929 media, for 7 d. After differentiation, macrophages were pre-treated with DMSO (# BP231-100; Thermo Fisher Scientific), FBX (200 µM; #S1547; Selleck Chemicals), Allo (250 μg/ml; #A8003; Sigma-Aldrich), or Casp-i (Z-YVAD-FMK, 20 μM; #218746; Sigma-Aldrich) for 30 min, and then with LPS (100 ng/ml; #L2630; Sigma-Aldrich) and IFNγ (20 ng/ml; #315-05; PeproTech), according to figure legends. In experiments in which cytokine secretion was measured, ATP (1 mM; #A6419; Sigma-Aldrich) was added 3 h before harvest. Supernatants were collected and kept at −80°C until further analysis.

### Cytokine release measurement

Supernatants were thawed and cytokine levels measured by ELISA (BioLegend) according to the manufacturer’s instructions (mouse TNF-α, Cat #430904; mouse IL-6, Cat #431304; mouse IL-1β, Cat #432604; human TNF-α, Cat #430204; human IL-6, Cat #430504; human IL-1β, Cat #437004).

### Caspase-1 activity assay

Macrophages were seeded at 5 × 10^4^ cells per condition in 384-well white plates and treated as described above. After 6-h incubation with LPS and IFNγ, assay buffer was added to each well (1:1 ratio) and caspase-1 activity was measured using Caspase-Glo 1 Inflammasome Assay (#G9951; Promega), according to the manufacturer’s instructions.

### Flow cytometry

BMDMs were plated at 2.5 × 10^5^ cells per condition and pre-treated for 30 min with vehicle (DMSO), Fbx (200 μM), Allo (250 μg/ml), or Casp-i (20 μM) followed by polarization with LPS (100 ng/ml) and IFNγ (20 ng/ml) for 18 h. After removal of the supernatant, cells were harvested in cold FACS buffer (PBS + 2% FBS) using a cell scraper and washed once in FACS buffer by centrifugation at 400*g* for 5 min. Cells were then resuspended in appropriate antibody cocktail (Table 1) and incubated at 4°C in the dark for 20 min. Finally, BMDMs were washed once more and resuspended in FACS buffer, and acquired using a BD FACS Symphony A1. Data were analysed using FlowJo software (BD).

**Table 1.** Flow cytometry antibodies.

Surface markers	Fluorochrome	Clone	Cat #	Supplier
Fixable viability dye	eF780	N/A	65-0865-18	Thermo Fisher Scientific
F4/80	Sb702	BM8	67-4801-82	Thermo Fisher Scientific
CD80	BV510	16-10A1	104741	BioLegend
CD86	AF700	GL-1	105024	BioLegend
CellROX	DeepRed	N/A	C10422	Thermo Fisher Scientific
MitoSOX	Red	N/A	M36008	Thermo Fisher Scientific
Intracellular markers				
iNOS	AF647	C-11	sc-727	Santa Cruz Biotechnology
pro-IL-1β	FITC	NJTEN3	11-7114-82	Thermo Fisher Scientific

### Intracellular staining

Cells were fixed and permeabilized using Cytofix/Cytoperm kit (#00-551-00; eBioscience) according to the manufacturer’s instructions. Cells were then washed in Perm buffer (#00-8333-56; Invitrogen) by centrifugation at 400*g* for 5 min and resuspended in appropriate antibody cocktail (Table 1) prepared in Perm buffer and incubated at 4°C in the dark for 20 min. BMDMs were then washed once more in Perm buffer and resuspended in FACS buffer for acquisition using a BD FACS Symphony A1. Data were analysed using FlowJo software (BD).

### ROS measurements

Cellular and mitochondrial ROS measurements were done using CellROX (#C10422; Thermo Fisher Scientific) and MitoSOX (#M36008; Thermo Fisher Scientific), respectively. For this, BMDMs were harvested using a cell scraper and washed in pre-warmed FACS buffer. Surface staining antibody cocktail containing ROS measurement dye was prepared using pre-warmed FACS buffer and, after cell resuspension, added to cells at 37°C for 15 min (MitoSOX) or 30 min (CellROX). BMDMs were washed and resuspended in pre-warmed FACS buffer and immediately acquired using a BD FACS Symphony A1. Data were analysed using FlowJo software (BD).

### Caspase inhibition screening assay

Caspase inhibitor (Z-YVAD-FMK), Fbx, and Allo were diluted for final concentrations ranging from 10^−10^ to 10^−6^ M. Caspase-1 inhibition was measured according to the manufacturer’s instructions (#701840; Cayman Chemicals). Relative inhibition was compared with the internal control of the assay, z-YVAD-CHO.

### qRT-PCR

RLT lysis buffer (QIAGEN) supplemented with 1% β-mercaptoethanol (Sigma-Aldrich) was added directly to the cell culture plates. Lysates were harvested, and RNA was extracted using RNeasy extraction kits (#74134; QIAGEN), according to the manufacturer’s instructions. RNA was quantified using a NanoDrop, and reverse transcription was executed at 50 ng/μl using High-Capacity cDNA Reverse Transcription Kit (#4374966; Applied Biosystems), according to the manufacturer’s instructions. qRT-PCR was performed using PowerTrack SYBR Green Master Mix (#A46111; Applied Biosystems), according to the manufacturer’s instructions, and ViiA 7 Real-Time PCR System (Thermo Fisher Scientific). All primers (Table 2) were validated for linearity and efficiency, and relative expression was determined accordingly.

**Table 2.** qRT- PCR primers.

Genes (mouse)	Forward sequence (5′-3′)	Reverse sequence (5′-3′)	Efficiency
*Il1b*	TGGACCTTCCAGGATGAGGACA	GTTCATCTCGGAGCCTGTAGTG	92%
*Tnfa*	GGTGCCTATGTCTCAGCCTCTT	GCCATAGAACTGATGAGAGGGAG	96%
*Il6*	TACCACTTCACAAGTCGGAGGC	CTGCAAGTGCATCATCGTTGTTC	92%
*18s*	GGGAGAGGAGCGAGCGACCA	CGCACGGCCGGTACAGTTAA	98%
*Rplp0*	TTATAACCCTGAAGTGCTCGAC	CGCTTGTACCCATTGATGATG	101%

### LC-MS

Cells were washed once with cold PBS. Cold (−20°C) methanol:acetonitrile:water (50:30:20) was added followed by cell harvest. Cells were spun down, and the metabolite extract (supernatant) was collected. Cold (−20°C) methanol:acetonitrile:water (50:30:20) was added to the residual cells and spun down, and the metabolite extract was collected a second time. Metabolite extracts were dried using a SpeedVac and stored at −80°C until further analysis.

For LC-MS analysis, dried extracts were resuspended in 50 µl of 20:80 methanol:water, spun down to remove debris, and sent out for LC-MS analysis at the Proteomics Core Facility of the Life Sciences Institute at the University of British Columbia. Samples were analysed by hydrophilic interaction liquid chromatography coupled to high-resolution tandem mass spectrometry (HILIC-HRMS/MS), following a previously described method (Qu et al, 2024) with adaptations to improve performance. Chromatographic separation was performed on an InfinityLab Poroshell 120 HILIC-Z column (2.7 μm, 150 × 2.1 mm; Agilent Technologies) using a Vanquish UHPLC system (Thermo Fisher Scientific), coupled to an IMPACII high-resolution mass spectrometer (Bruker Daltonics). The mobile phases consisted of (A) water with 0.1% (vol/vol) formic acid, 10 mM ammonium acetate, and 2 μM medronic acid, and (B) 90% (vol/vol) acetonitrile with 10% (vol/vol) water, supplemented with the same modifiers. A multi-step gradient was applied: starting with 90% B, held for 2 min, followed by a linear decrease to 40% B over 6 min, maintained at 40% B for 2 min, returned to 90% B over 1 min, and re-equilibrated for 5 min. The total run time was 16 min. The flow rate was 0.3 ml/min, the column was maintained at 30°C, and the autosampler was kept at 4°C. Mass spectrometry data were acquired in both positive (ESI^+^) and negative (ESI^−^) electrospray ionization modes using data-dependent acquisition (DDA) to collect both precursor and fragment ion information. To enhance structural elucidation and minimize redundant fragmentation, a 15-s active exclusion window was applied, improving isomer coverage. For ESI^−^ mode, instrument settings were as follows: capillary voltage at −3,800 V, nebulizer gas pressure at 2.0 bar, dry gas flow rate at 9 litres min^−1^, dry gas temperature at 220°C, and a mass scan range of 100–1,300 m/z. Spectra were acquired at 2 Hz with a cycle time of 0.6 s. Collision energy was ramped from 20 V to 250% throughout each MS/MS scan. For ESI^+^ acquisitions, the capillary voltage was set to 4,500 V, whereas all other settings remained the same as in negative mode. Internal calibration was performed for each sample by delivering 10 mM sodium formate into the void volume via a six-port diverter valve, achieving mass accuracy below 2 ppm. A quality control (QC) sample was injected every eight samples to monitor system performance and reproducibility. The following *m/z* values were tracked (Table 3).

**Table 3.** LC-MS metabolites.

Metabolite	Retention time (min)	*m/z*
Succinate	1.12	117.0192 (negative mode)
Itaconate	1.09	153.0158 (positive mode, m+Na)
Hypoxanthine	1.20	159.0278 (positive mode, m+Na)
Xanthine	1.12	151.0258 (negative mode)

### Uric acid levels

Cells were harvested using cold PBS + 2.5 mM ETDA. After cell scraping, cells were washed 1X with cold PBS and suspended in the assay buffer. Uric acid levels were measured using the commercially available uric acid detection kit (#AB65344; Abcam), according to the manufacturer’s instructions.

### Fluorescent microscopy

Cells were washed once with PBS and fixed with 4% PFA for 15 min at room temperature in the dark. Cells were then washed with PBS and blocked with 5% milk in TBS+0.1% Tween. Samples were rinsed with TBS+0.1% Tween, and primary antibodies (in 5% milk in TBS+0.1% Tween; mouse ASC, #67824; Cell Signaling, human ASC, #13833; Cell Signaling, NLRP3, #15101; Cell Signaling) were added for 1 h at room temperature. Samples were rinsed with TBS+0.1% Tween and incubated with secondary antibodies for 1 h at room temperature in the dark (anti-rabbit IgG F(ab’)_2_ Fragment Alexa Fluor 488 Conjugate, #4412; Cell Signaling, anti-rabbit IgG F(ab’)_2_ Fragment Alexa Fluor 647 Conjugate, #4414; Cell Signaling). Samples were rinsed with TBS+0.1% Tween, and DAPI (1 μg/ml; #4083; Cell Signaling) was added for 5 min. Samples were rinsed and resuspended with PBS for image acquisition using the Keyence BZ-X810 fluorescence microscope. Figures and quantifications were generated with ImageJ (Fiji) software.

### NAD/NADH

Macrophages were seeded at 5 × 10^4^ cells per condition in 384-well white plates. NAD+ and NADH levels were measured using NAD/NADH-Glo Assay according to the manufacturer’s instructions (#G9071; Promega).

### Human ethics

The study was approved by the BC Children’s Hospital Research Institute ethics committee (protocol #H19-02734), and all healthy adult donors signed a consent form.

### Human MDM

Peripheral blood (100 ml) was collected from healthy donors in K3-EDTA tubes (Cat #95057-237; VACUETTE). Blood was diluted 1:1 in PBS, and mononuclear leucocytes were collected using gradient centrifugation (Lymphoprep #07861; STEMCELL Technologies). Residual red blood cells were lysed using red blood cell lysis buffer for 5 min. Mononuclear leucocytes were then plated in RPMI containing 10% FBS and 1% Pen/Strep for 3 h. Non-adherent cells were removed, and remaining monocytes were treated with human M-CSF (50 ng/ml; #300-25; PeproTech) for 7 d. Monocyte-derived macrophages were then pre-treated for 30 min with DMSO, Fbx (200 µM), or Allo (250 μg/ml), and then with LPS (100 ng/ml) and human IFNγ (20 ng/ml; #300-02; PeproTech), according to figure legends. In experiments in which cytokine release was measured, ATP (1 mM) was added 3 h before harvesting.

### Software

All statistical analysis was generated using Prism 10 (GraphPad Software). Schematics were generated using BioRender. Flow cytometry plots were analysed with FlowJo software (BD). Image quantifications were generated with ImageJ (Fiji).

## Supplementary Material

Reviewer comments

## Data Availability

Further information and requests for resources and reagents should be directed to and will be made available upon reasonable request by the Lead Contact, RI Klein Geltink (ramon.kleingeltink@bcchr.ca).
