# Supplementary material for: Inhibition of xanthine oxidoreductase with febuxostat, but not allopurinol, prevents inflammasome assembly and IL-1β release
Source: Life Sci Alliance. 2025 May 21;8(8):e202403191. doi: 10.26508/lsa.202403191 (PMC12095928; doi:10.26508/lsa.202403191)
Supplement: Reviewer comments [file LSA-2024-03191_review_history.pdf]

Life Science Alliance

Inhibition of xanthine oxidoreductase with febuxostat, but not allopurinol, prevents inflammasome assembly and IL-1β release

Lauar de Brito Monteiro, Anne-Sophie Archambault, Lucas F Starchuk, Armando Alcazar, Ju Hee Oh, Joshua A Dubland, Bojana Rakić, Annette E Patterson, Dr. C. Bruce Verchere and Ramon I. Klein Geltink
DOI: https://doi.org/10.26508/lsa.202403191

Corresponding author(s): Dr. Ramon I Klein Geltink (University of British Columbia)

Review Timeline:

Submission Date:	2024-12-24
Editorial Decision:	2025-02-28
Revision Received:	2025-04-02
Editorial Decision:	2025-04-21
Revision Received:	2025-04-30
Accepted:	2025-05-14

Scientific Editor: Tim Fessenden, PhD

Transaction Report:

(Note: With the exception of the correction of typographical or spelling errors that could be a source of ambiguity, letters and reports are not edited. The original formatting of letters and referee reports may not be reflected in this compilation.)

No full Peer Review Process File is available with this article, as the authors have chosen not to make the review process public in this case.

February 28, 2025

Re: Life Science Alliance manuscript #LSA-2024-03191-T

Dr. Ramon I Klein Geltink
University of British Columbia
Pathology and Laboratory Medicine
928 W 28th Ave
Room A4-148
Vancouver, BC V5Z 4H4
Canada

Dear Dr. Klein Geltink,

Thank you for submitting your manuscript entitled "Inhibition of xanthine oxidoreductase with febuxostat, but not allopurinol, prevents inflammasome assembly and IL-1β release" to Life Science Alliance. The manuscript was assessed by expert reviewers, whose comments are appended to this letter. We invite you to submit a revised manuscript addressing the Reviewer comments.

To upload the revised version of your manuscript, please log in to your account: https://lsa.msubmit.net/cgi-bin/main.plex

You will be guided to complete the submission of your revised manuscript and to fill in all necessary information. Please get in touch in case you do not know or remember your login name.

While you are revising your manuscript, please also attend to the below editorial points to help expedite the publication of your manuscript. Please direct any editorial questions to the journal office.

The typical timeframe for revisions is three months. Please note that papers are generally considered through only one revision cycle, so strong support from the referees on the revised version is needed for acceptance.

When submitting the revision, please include a letter addressing the reviewers' comments point by point.

We hope that the comments below will prove constructive as your work progresses.

Thank you for this interesting contribution to Life Science Alliance. We are looking forward to receiving your revised manuscript.

Sincerely,

Eric Sawey, PhD
Executive Editor
Life Science Alliance
http://www.lsajournal.org

A. THESE ITEMS ARE REQUIRED FOR REVISIONS

-- A letter addressing the reviewers' comments point by point.

-- An editable version of the final text (.DOC or .DOCX) is needed for copyediting (no PDFs).

-- High-resolution figure, supplementary figure and video files uploaded as individual files: See our detailed guidelines for preparing your production-ready images, https://www.life-science-alliance.org/authors

-- Summary blurb (enter in submission system): A short text summarizing in a single sentence the study (max. 200 characters including spaces). This text is used in conjunction with the titles of papers, hence should be informative and complementary to the title and running title. It should describe the context and significance of the findings for a general readership; it should be written in the present tense and refer to the work in the third person. Author names should not be mentioned.

-- By submitting a revision, you attest that you are aware of our payment policies found here: https://www.life-science-alliance.org/copyright-license-fee

B. MANUSCRIPT ORGANIZATION AND FORMATTING:

Full guidelines are available on our Instructions for Authors page, https://www.life-science-alliance.org/authors

We encourage our authors to provide original source data, particularly uncropped/-processed electrophoretic blots and spreadsheets for the main figures of the manuscript. If you would like to add source data, we would welcome one PDF/Excel-file per figure for this information. These files will be linked online as supplementary "Source Data" files.

IMPORTANT: It is Life Science Alliance policy that if requested, original data images must be made available. Failure to provide original images upon request will result in unavoidable delays in publication. Please ensure that you have access to all original microscopy and blot data images before submitting your revision.

April 21, 2025

RE: Life Science Alliance Manuscript #LSA-2024-03191-TR

Dr. Ramon I Klein Geltink
University of British Columbia
Pathology and Laboratory Medicine
928 W 28th Ave
Room A4-148
Vancouver, BC V5Z 4H4
Canada

Dear Dr. Klein Geltink,

Thank you for submitting your revised manuscript entitled "Febuxostat, but not allopurinol, prevents inflammasome assembly and IL-1β release from macrophages". As you will see, reviewers are now satisfied. We would be happy to publish your paper in Life Science Alliance pending final revisions necessary to meet our formatting guidelines, noted below.

In the Methods section, please include full details of mass spectrometry performed by the Proteomics Core Facility of the Life Sciences Institute.

Along with points mentioned below, please tend to the following:
-please be sure that the authorship listing and order is correct.
-please add the X and Bluesky handles of your host institute/organization as well as your own or/and one of the authors in our system.
-titles in the manuscript submission system and manuscript file must match.
-please incorporate any points from the Conclusion section into the Discussion; we only allow a Discussion section.
-please add an Author Contributions section to your main manuscript text.
-please add a Conflict of Interest statement to your main manuscript text.
-please use the [10 author names, et al.] format in your references (i.e., limit the author names to the first 10).
-please add your main, supplementary figure, and table legends to the main manuscript text after the references section.
-since figure S2 has only one panel, it is unnecessary to label it as A. Please remove it from the figure and call-out in the manuscript text.
-please remove figures from the manuscript text and leave them uploaded separately.

If you are planning a press release on your work, please inform us immediately to allow informing our production team and scheduling a release date.

LSA now encourages authors to provide a 30-60 second video where the study is briefly explained. We will use these videos on social media to promote the published paper and the presenting author (for examples, see https://docs.google.com/document/d/1-UWCfbE4pGcDdcgzcmiuJl2XMBJnxKYeqRvLLrLSo8s/edit?usp=sharing). Corresponding or first-authors are welcome to submit the video. Please submit only one video per manuscript. The video can be emailed to contact@life-science-alliance.org

To upload the final version of your manuscript, please log in to your account: https://lsa.msubmit.net/cgi-bin/main.plex
You will be guided to complete the submission of your revised manuscript and to fill in all necessary information. Please get in touch in case you do not know or remember your login name.

To avoid unnecessary delays in the acceptance and publication of your paper, please read the following information carefully.

A. FINAL FILES:

These items are required for acceptance.

-- An editable version of the final text (.DOC or .DOCX) is needed for copyediting (no PDFs).

-- High-resolution figure, supplementary figure and video files uploaded as individual files: See our detailed guidelines for preparing your production-ready images, https://www.life-science-alliance.org/authors

-- Summary blurb (enter in submission system): A short text summarizing in a single sentence the study (max. 200 characters including spaces). This text is used in conjunction with the titles of papers, hence should be informative and complementary to the title. It should describe the context and significance of the findings for a general readership; it should be written in the present tense and refer to the work in the third person. Author names should not be mentioned.

B. MANUSCRIPT ORGANIZATION AND FORMATTING:

Full guidelines are available on our Instructions for Authors page, https://www.life-science-alliance.org/authors

We encourage our authors to provide original source data, particularly uncropped/-processed electrophoretic blots and spreadsheets for the main figures of the manuscript. If you would like to add source data, we would welcome one PDF/Excel-file per figure for this information. These files will be linked as supplementary "Source Data" files.

Submission of a paper that does not conform to Life Science Alliance guidelines will delay the acceptance of your manuscript.

It is Life Science Alliance policy that if requested, original data images must be made available to the editors. Failure to provide original images upon request will result in unavoidable delays in publication. Please ensure that you have access to all original data images prior to final submission.

The license to publish form must be signed before your manuscript can be sent to production. A link to the license to publish form will be available to the corresponding author only. Please take a moment to check your funder requirements.

Reviews, decision letters, and point-by-point responses associated with peer-review at Life Science Alliance will be published, alongside the manuscript. If you do want to opt out of having the reviewer reports and your point-by-point responses displayed, please let us know immediately.

Thank you for your attention to these final processing requirements. Please revise and format the manuscript and upload materials within 7 days.

Thank you for this interesting contribution, we look forward to publishing your paper in Life Science Alliance.

Sincerely,

Tim Fessenden
Scientific Editor
Life Science Alliance

May 14, 2025

RE: Life Science Alliance Manuscript #LSA-2024-03191-TRR

Dr. Ramon I Klein Geltink
University of British Columbia
Pathology and Laboratory Medicine
928 W 28th Ave
Room A4-148
Vancouver, BC V5Z 4H4
Canada

Dear Dr. Klein Geltink,

Thank you for submitting your Research Article entitled "Inhibition of xanthine oxidoreductase with febuxostat, but not allopurinol, prevents inflammasome assembly and IL-1β release". It is a pleasure to let you know that your manuscript is now accepted for publication in Life Science Alliance. Congratulations on this interesting work.

The final published version of your manuscript will be deposited by us to PubMed Central upon publication.

Your manuscript will now progress through copyediting and proofing. It is journal policy that authors provide original data upon request.

Reviews, decision letters, and point-by-point responses associated with peer-review at Life Science Alliance will be published, alongside the manuscript. If you do want to opt out of having the reviewer reports and your point-by-point responses displayed, please let us know immediately.

IMPORTANT: If you will be unreachable at any time, please provide us with the email address of an alternate author. Failure to respond to routine queries may lead to unavoidable delays in publication.

Scheduling details will be available from our production department. You will receive proofs shortly before the publication date. Only essential corrections can be made at the proof stage so if there are any minor final changes you wish to make to the manuscript, please let the journal office know now.

DISTRIBUTION OF MATERIALS:
Authors are required to distribute freely any materials used in experiments published in Life Science Alliance. Authors are encouraged to deposit materials used in their studies to the appropriate repositories for distribution to researchers.

You can contact the journal office with any questions, contact@life-science-alliance.org

Again, congratulations on a very nice paper. I hope you found the review process to be constructive and are pleased with how the manuscript was handled editorially. We look forward to future exciting submissions from your lab.

Sincerely,

Tim Fessenden
Scientific Editor
Life Science Alliance--
